# Chronobiology and Metabolism: Is Ketogenic Diet Able to Influence Circadian Rhythm?

**DOI:** 10.3389/fnins.2021.756970

**Published:** 2021-11-08

**Authors:** Elena Gangitano, Lucio Gnessi, Andrea Lenzi, David Ray

**Affiliations:** ^1^Department of Experimental Medicine, Sapienza University of Rome, Rome, Italy; ^2^Oxford Centre for Diabetes, Endocrinology and Metabolism, University of Oxford, Oxford, United Kingdom; ^3^NIHR Oxford Biomedical Research Centre, John Radcliffe Hospital, Oxford, United Kingdom

**Keywords:** chronobiology, circadian rhythm, circadian misalignment, metabolism, ketogenic diet (KD)

## Abstract

Circadian rhythms underpin most physiological processes, including energy metabolism. The core circadian clock consists of a transcription-translation negative feedback loop, and is synchronized to light-dark cycles by virtue of light input from the retina, to the central clock in the suprachiasmatic nucleus in the hypothalamus. All cells in the body have circadian oscillators which are entrained to the central clock by neural and humoral signals. In addition to light entrainment of the central clock in the brain, it now emerges that other stimuli can drive circadian clock function in peripheral tissues, the major one being food. This can then drive the liver clock to be misaligned with the central brain clock, a situation of internal misalignment with metabolic disease consequences. Such misalignment is prevalent, with shift workers making up 20% of the working population. The effects of diet composition on the clock are not completely clarified yet. High-fat diet and fasting influence circadian expression of clock genes, inducing phase-advance and phase-delay in animal models. Ketogenic diet (KD) is able to induce a metabolic switch from carbohydrate to fatty acid oxidation, miming a fasting state. In recent years, some animal studies have been conducted to investigate the ability of the KD to modify circadian gene expression, and demonstrated that the KD alters circadian rhythm and induces a rearrangement of metabolic gene expression. These findings may lead to new approaches to obesity and metabolic pathologies treatment.

## Introduction

### The Clock Machinery and the Circadian Rhythm

In mammalians many genes exhibit daily fluctuations in their expression levels, configuring a circadian rhythm of approximately 24 h. This circadian oscillation is internally generated (Ko and Takahashi, 2006; Dibner et al., 2010) and driven by clock machinery.

At a cellular level the circadian clock is constituted by core clock genes, including *CLOCK*, *BMAL1*, *PER*, and *CRY*, that are connected by transcriptional-translational feedback loops (Albrecht, 2012). The feedback loop produces oscillations in gene expression, associated with circadian changes in chromatin architecture, mRNA processing, and protein activity and turnover (Eckel-Mahan et al., 2013). CLOCK and BMAL1 are the core heterodimeric transcription factor which drives expression of *PER*, and *CRY* genes, and the *REVERB* genes, by binding to conserved DNA sequences termed E boxes. BMAL1/CLOCK transactivation of target genes such as *PER* and *CRY*, which are part of the negative feedback loop, results in the accumulation of their protein products PER and CRY proteins. PER and CRY act together to repress the activity of the BMAL1/CLOCK heterodimer, thus forming one negative feedback loop. CLOCK:BMAL1 also activate the transcription of the nuclear receptors REV-ERB and RORA, whose proteins compete to bind the *BMAL1* promoter at a ROR binding site. ROR activates *BMAL1* transcription, while REV-ERBs represses it (Ko and Takahashi, 2006). Thus there are two negative feedback loops that together confer a robust 24 h-period oscillator.

**FIGURE 1:**
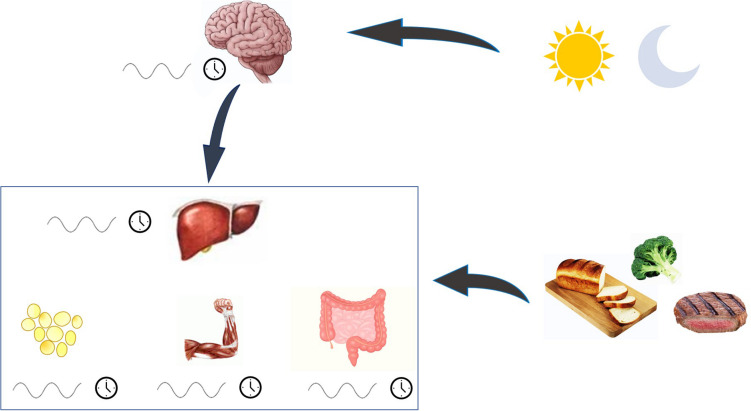
The regulation of circadian rhythm. Biological rhythm is under the control of the central clock, set in the suprachiasmatic nucleus. This clock integrates its internal rhythm with the alternance of light and dark, and orchestrates the rhythm of peripheral clocks, which are directly entrained also by food. When all clocks are synchronized, metabolism functioning is preserved.

The circadian clock synchronizes internal metabolism with the environment, interacting with light-dark and feeding-fasting state (Panda, 2016). The central clock is set in the suprachiasmatic nucleus (SCN) of the anterior hypothalamus, and it is synchronized to the external light-dark cycle by direct neural input from the retina. The central clock is set at the top of the hierarchy of the circadian oscillation and drives the peripheral clocks oscillation in a synchronized way. In peripheral tissues all cells also have a circadian oscillator. However, in the absence of light-sensing these clocks are kept synchronized by neural and humoral outputs from the SCN. In addition, peripheral clocks will entrain to feeding cycles, a phenomenon termed food entrainment (see Figure 1). It is possible to drive the circadian phase of the liver to be in anti-phase to that of the SCN by restricting feeding to the conventional rest period (Damiola et al., 2000); night in humans. Other metabolic tissues, as adipose tissue, have their own clocks, which are normally kept synchronized with the liver clock and the other peripheral clocks.

The clock machinery controls physiology processes by regulating the expression of hundreds of metabolic genes, involved in the rate-limiting steps of fundamental metabolic pathways. Therefore it orchestrates metabolism in cycles of 24 h, and increases metabolic efficiency thanks to anticipatory responses to the feeding-fasting cycles and temporal separation of opposite metabolic processes (Panda, 2016; Poggiogalle et al., 2018; Reinke and Asher, 2019).

Peripheral tissues are implicated in the metabolic balance of the individual. Among them, the liver plays a key role in major enzymatic processes for glucose and fat metabolism. Therefore, the SCN-liver axis for circadian rhythm is probably the most important connection between the central clock and the peripheral clocks.

### The Ketogenic Diet

Ketogenic Diet (KD) is a low-carbohydrate, normo-protein diet, characterized by an overproduction of the ketone bodies acetone, acetoacetate and β-hydroxyl-butyrate (βOHB). KD induces a metabolic switch from carbohydrate to fatty acid oxidation, so that fat is the principal energy source. During a KD, fatty acid oxidation, ketogenesis and gluconeogenesis are upregulated and glycolysis and *de novo* lipogenesis are markedly reduced (Tognini et al., 2017), and these metabolic modifications are similar to those physiologically seen during fasting or significative caloric restriction (Tognini et al., 2017). Ketone bodies are not only an energy source, directed from the liver to the periphery during fasting conditions and exercise, but also important signaling molecules (Newman and Verdin, 2014). βOHB can bind to G-protein-coupled receptors for short-chain fatty acids on the cell surface, reducing lipolysis (Taggart et al., 2005) and sympathetic activity (Kimura et al., 2011). On the other hand, short-chain fatty acids promote sympathetic nervous system activation (Kimura et al., 2011). Ketone bodies are also able to inhibit histone deacetylase and induce hyperacetylation, similarly to what happens in fasting conditions, and determine changes in gene expression (Shimazu et al., 2013; Newman and Verdin, 2014). In addition, ketone bodies are also implicated in the mechanism of food anticipation (Chavan et al., 2016; Chaix and Panda, 2016).

In clinical settings, there are different kinds of KD, which differ in the degree of calorie restriction and macronutrient composition (Kirkpatrick et al., 2019). The KD was originally proposed for the treatment of refractory epilepsy in children and over time has been proven to be particularly effective in treating morbid obesity and metabolic diseases, providing a relatively fast weight loss with concomitant preservation of muscle mass. KD characterized by a very low-calorie content (VLCKD) is actually considered for prescription in severe obesity and obesity complicated by type 2 diabetes, hypertriglyceridemia and/or hypertension (Caprio et al., 2019), that configure a picture of severely metabolically compromised patients. There are some contraindications to KD prescription. In particular, according to the Italian consensus on VLCKD, it is absolutely contraindicated in some patients, as patients with organ failure (respiratory failure, kidney failure and moderate-to-severe chronic kidney disease, hepatic failure), some cardiovascular diseases (heart failure, unstable angina, cardiac arrhythmias and recent stroke or myocardial infarction), severe infections, type 1 diabetes mellitus, beta-cell failure in type 2 diabetes mellitus and therapy with sodium/glucose cotransporter-2 inhibitors. Moreover, VLCKD is contraindicated in frail elderly patients, during pregnancy and breastfeeding, in the peri-operative period and in case of concomitant psychiatric conditions (severe mental illnesses, eating disorders, alcohol and substance abuse) (Caprio et al., 2019).

Ketogenic diet has been recently proposed as an adjuvant treatment for other illnesses (Paoli et al., 2013), as migraine (Di Lorenzo et al., 2019), polycystic ovary syndrome (Paoli et al., 2020), cancer (Chung and Park, 2017), neurodegenerative diseases (Wlodarek, 2019) and even COVID-19 (Gangitano et al., 2021; Sukkar et al., 2021). Question remains about the relative impact of fat content, protein content, and overall calorie content.

## Clock Desynchronization and Metabolism: The Importance of the Timing of Food Intake, Fasting and Sleep

**FIGURE 2:**
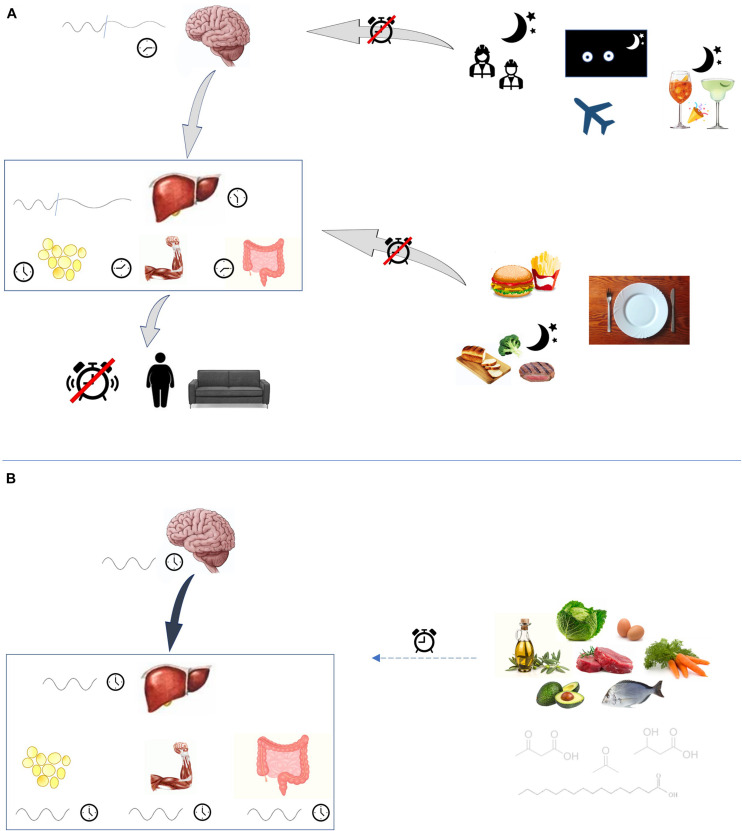
Circadian rhythm disruption. **(A)** Many external factors may disturb circadian rhythmicity. Shift-work, jet-lag and social jet-lag alter the exposition to light and dark cycles. Also lack of sleep and low-quality sleep are able to disrupt central clock rhythm. Consumption of high-fat foods, as well as eating at night-time and fasting, have disruptive effects on peripheral clocks, which are led out of phase. Clocks desynchronization favors the onset of metabolic disturbances. **(B)** Ketogenic diet, probably through its peculiar molecules fatty acids and ketone bodies, may play a role in influencing circadian rhythm.

Internal misalignment between the phase of the circadian cycle in the central clock and that of the peripheral clocks, results in disorder amongst the organs implicated in metabolism, and behavior, and may drive the onset of disease. Indeed, this prevalent phenomenon occurs in shift workers who have an increased risk of obesity and type 2 diabetes (see Figure 2).

Clock-mutant mice, with preserved rhythmicity in the suprachiasmatic nucleus and pineal gland, and arrhythmic clock genes expression in liver and skeletal muscle, are metabolically impaired, with altered glucose tolerance and insulin secretion, and altered expression of molecular determinants of metabolic homeostasis in liver and skeletal muscle, in comparison with wild-type mice (Kennaway et al., 2007).

Eating against the clock drives internal desynchronization and, among peripheral clocks, the hepatic clock is particularly sensitive to feeding time information (Damiola et al., 2000; Eckel-Mahan et al., 2013). Studies in mice reveal that animals fed during the resting phase gain weight, develop abdominal obesity, metabolic alterations (Salgado-delgado et al., 2010; Yasumoto et al., 2016), increased hepatic fat accumulation, hyperphagia and are less physically active (Yasumoto et al., 2016). These metabolically impaired animals develop alterations in the circadian expression of clock genes (Turek et al., 2005; Salgado-Delgado et al., 2013; Yasumoto et al., 2016), confirming the strict association between circadian rhythm and metabolism. Feeding during the resting phase leads to desynchronization among liver and skeletal muscle peripheral clocks (Yasumoto et al., 2016), desynchronization among liver clock genes and metabolic genes expression (Salgado-Delgado et al., 2013) and alteration of the expression of glucose and lipid metabolism-related genes (Yasumoto et al., 2016). The disruption of circadian rhythm selectively hits peripheral clocks, as the central clock is only affected by light information (Damiola et al., 2000).

By contrast, feeding mice during the active phase prevents metabolic alterations and clock genes disruption, despite the night-shift work (Salgado-delgado et al., 2010) and the diet composition (Hatori et al., 2012). Studies on mice fed High-Fat (HF) diet with time-restricted access to food of 8 h/day, during the natural feeding time, in comparison with mice fed HF *ad libitum*, showed that, given the same caloric intake, weight gain and metabolic disturbances were restricted to the ad-lib fed animals, and that time-restricted feeding prevented the metabolic consequences of an unhealthy diet (Hatori et al., 2012). In fact, restricting food consumption to the active phase led to increased amplitude of the circadian oscillation in peripheral tissues and it was not associated to the development of obesity, hyperinsulinemia and hepatic steatosis (Hatori et al., 2012; Bae et al., 2019).

Intermittent fasting, with food given every other day, has been shown to be able to abolish the circadian expression of most clock genes, while *Clock* and *Per2* were expressed with a decreased amplitude and a phase-advance in mouse liver (Froy et al., 2009). On the contrary, intermittent fasting was able to restore circadian rhythms and clock gene expression amplitude that was disrupted by abnormal light cycles, including cycles with a period of less than 24 h (Froy et al., 2009).

In mice without a central clock and subject to environmental disruption by being in constant darkness there is the complete disintegration of consolidated sleep-wake, and activity rhythms, with loss of feed/fasted cycles. Time-restricted food availability was sufficient to restore circadian organization to the animals’ behavior, and energy metabolism with normalization of body weight and glucose metabolism (Kolbe et al., 2019). Therefore, intermittent fasting seems to be able to affect circadian rhythm differently, depending on the time of food availability (Froy et al., 2009). Furthermore, complete fasting modulates the circadian rhythm by attenuating clock gene oscillation amplitude and also induces a circadian phase advance (Barnea et al., 2009). A simultaneous shift in feeding schedule and light and dark cycle has been proposed to facilitate circadian resetting in animal models (Wu et al., 2010).

In humans, the key role of the timing of food intake is confirmed by the fact that late eating is associated with increased BMI (Mchill et al., 2017, 2019), reduced insulin sensitivity and reduced effects of weight-loss strategies (Dashti et al., 2021). Breakfast consumption has been proven to affect clock and clock-controlled gene expression, and skipping breakfast alters core clock gene expression and also increases the postprandial glycemic response in both healthy and type 2 diabetes patients (Jakubowicz et al., 2017).

In people with sleep deprivation, typical of shift-workers, there is a preference for high-fat and sweet foods (Cain et al., 2015; Simon et al., 2015), and an association, which may be causal, with weight gain (Van Den Berg et al., 2008; Chaput et al., 2014) and development of obesity (Potter et al., 2017) and metabolic disturbances, as altered glucose tolerance (Grant et al., 2017; McHill and Wright, 2017). Even sleep fragmentation is associated with obesity (Van Den Berg et al., 2008; Mezick et al., 2014) and insulin resistance (McHill and Wright, 2017). A possible strategy to mitigate the risks of shiftwork is to avoid eating against the clock (Grant et al., 2017).

Social jet lag is a recently emerged phenomenon related to the shift of social life toward nocturnal hours, so that people meet each other and eventually eat and/or drink during hours normally dedicated to rest and sleep. It is characterized by a misalignment between social life and biological rhythms, configures a situation similar to the classic jet lag, and, like long flights, leads to altered circadian rhythm and metabolic consequences long-term (Almoosawi et al., 2018; Mathew et al., 2020).

## Clock Desynchronization and Metabolism: The Importance of Diet Composition

Diet composition can influence circadian clock activity, because feeding-derived metabolites play a role in regulating cellular rhythmicity (Eckel-Mahan et al., 2013), but the mechanisms through which nutrition influences the circadian metabolome and consequently circadian rhythm have not yet been elucidated.

Some animal studies show that HF diet is able to disrupt circadian rhythm (Kohsaka et al., 2007; Barnea et al., 2010), inducing a phase shift (Barnea et al., 2009, 2010; Eckel-Mahan et al., 2013) and a loss of synchronization of gene expression among liver and fat tissue (Kohsaka et al., 2007). Kohsaka et al. (2007) fed mice with a regular chow or an HF diet for 6 weeks. After 7 days, before a significant weight gain, mice fed with HF diet reduced their overall physical activity and increased the food intake during the resting period. Core clock gene expression was not affected in the hypothalamus, but the amplitude of *Clock* and *Bmal1* expression was attenuated in fat and liver. Moreover, a loss of synchrony of gene expression of nuclear receptors and metabolic regulators was observed among peripheral tissues.

Barnea et al. (2009, 2010) studied mice fed an HF diet or a low-fat (LF) diet for 7 weeks, followed by a day of fasting. Clock genes oscillated in the liver, muscle and white adipose tissue (WAT), but fasting in the LF diet group led to an attenuation of clock gene amplitude. Fasting caused a circadian phase advance, but in contrast an HF diet induced a phase delay in circadian clock genes and resulting disruption of the circadian rhythmicity of the adiponectin component cascade. Diet-induced disruption in the circadian expression of adiponectin signaling components may result from metabolite regulation of peroxisome proliferator-activated receptors α and γ (PPARα and PPARγ) and mAMPK. These signaling cascades may allow feeding time to affect core circadian clock function. In turn, disruption of the clock may result in aberrant coordination of adiponectin synthesis and processing. This alteration in adiponectin signaling may be related to the development of metabolic impairment and the disruption of other clock-controlled mechanisms, as blood pressure and sleep/wake cycle, associated with metabolic syndrome.

Eckel-Mahan et al. (2013) studied mice and showed that diet composition itself is able to reprogram the clock. In particular, an HF diet profoundly reorganizes specific metabolic pathways, with a widespread remodeling of the liver clock, ablates some transcript and metabolite oscillations, generates new oscillating transcripts and, in contrast to Barnea (Barnea et al., 2009, 2010), observed that it induces a phase advance for many metabolites and oscillating transcripts. These changes are maintained on the diet and are reversible. In addition, Eckel-Mahan identified new oscillating gene transcripts in the liver that were only seen on the HF diet, while they observed that some typically oscillating genes lost a circadian signature. HF reorganizes coordinated oscillation of transcripts and metabolites through shifted CLOCK: BMAL1 chromatin recruitment and cyclic activation of surrogate pathways through the transcription factor PPARγ. *Clock* and *Bmal1* transcription, protein levels and their phosphorylation were unaltered in livers of HF-diet fed mice. Three days of HF diet were enough to initiate the reprogramming of the circadian clock, confirming the results of Kohsaka (Kohsaka et al., 2007) that an HF diet is able to alter circadian rhythm and behavioral activity independently from weight gain. Two weeks of normal chow were able to restore the circadian clock, proving that the transcriptional and epigenetic modifications induced by the HF diet are reversible.

## Circadian Rhythm and Ketogenic Diet

The effects of a ketogenic diet on clock gene expression have been recently investigated in mouse models, and these studies showed that KD is able to influence circadian rhythm.

Oishi et al. (2009, 2013) fed mice with KD or normal chow for 2 weeks, and observed that KD induced a phase-advance in peripheral clocks and behavioral activity, despite a maintained light-dark cycle and feeding *ad libitum*. The phase-advance effect was greater in the heart, kidney, and adipose tissue, than in the liver. Moreover, some clock genes showed a higher amplitude of their expression in the liver, while their amplitude in the heart was substantially unaffected (Oishi et al., 2013). The authors hypothesized that this robust oscillation may be related to CIRBP expression, a protein linked to hypothermia in mice (Oishi et al., 2013).

On the contrary, Genzer et al. (2015) fed mice with a LF diet or KD for 8 weeks, and observed that clock genes were phase-delayed under KD compared to the LF diet in the brain and the liver. Moreover, the amplitude of the circadian rhythm of clock genes in the liver was sixfold higher in the KD group, while their amplitude was lower in the brain, except for *Bmal1* that showed an increase in the amplitude of oscillation. The high-amplitude circadian rhythm in the periphery reflected an increased locomotor activity. In contrast to Oishi, their gene analysis was performed under conditions of total darkness, thereby avoiding light as a confounding factor.

Tognini et al. (2017) studied the molecular mechanisms which may lay behind the influence of KD on circadian rhythm. The authors fed mice *ad libitum* with normal chow or a KD for 4 weeks, and then studied the effects of the diets in the liver and the gut. With normal chow, the number of cyclic genes was similar in the liver and in the gut, while KD induced many *de novo* oscillating genes in the liver, and reduced the oscillating genes in the gut, revealing a tissue-specificity of the metabolic effects induced by the KD. Moreover, the diurnal KD-induced genes oscillated in a coordinated manner in the liver and in the gut. Among the genes oscillating both in normal chow and KD diet, more than a half had an increased amplitude in the liver under the KD diet. The expression of hepatic and gut core clock genes did not differ in mice fed the KD in comparison to normal chow-mice, so that core clock genes seem to be resistant to alterations induced by a food challenge. KD modulates the clock machinery recruitment to chromatin, leading to changes in the coupling of the core molecular clock machinery to output pathways. This is different to the HF diet which tends to hinder the chromatin recruitment of *Bmal1* (Eckel-Mahan et al., 2013). The ketone, βOHB, has an epigenetic role in histone post-translational modifications.

In humans there are no studies on the effects of a ketogenic diet on the circadian rhythm. Anyway sleep, as discussed above, is a direct expression of circadian rhythm. In the literature, there are some indirect data on the effects of KD on sleep. Diet composition can influence sleep quality and structure (Zadeh and Begum, 2011; Santana et al., 2012; Grandner et al., 2013; Tanaka et al., 2013; Yamaguchi et al., 2013; Katagiri et al., 2014; St-Onge et al., 2016a,b; Zhou et al., 2016; Komada et al., 2017). Studies on patients treated with KD show some interesting results on improving sleep structure. A study of 11 epileptic children administered KD for 12 months (Hallbook et al., 2007) showed a reduction of total sleep and daytime sleep, intact slow-wave sleep and an increase in rapid eye movement (REM) sleep, associated with improved attentional behavior. Similarly, morbidly obese adolescents which were administered a high-protein, low-carbohydrate, low-fat KD experienced increased REM and decreases slow-wave sleep from a supraphysiological level (Willi et al., 1998).

Afaghi et al. (2008) studied a sample of 14 healthy non-obese men administered a very low carbohydrate diet which induced ketosis, and an isocaloric control mixed diet, and observed increased slow-wave sleep and decreased REM sleep during the very low-carbohydrate diet administration. Castro et al. (2018) recently studied the effect of a very low-calorie, ketogenic diet on sleep in 20 obese patients, measuring the diurnal sleep propensity with the Epworth Daytime Sleepiness Scale (ESS) and the quality of sleep with the questionnaire Pittsburgh Sleep Quality Index (PSQI). The quantity and quality of sleep were reported as not changed during the administration of diet, over a time of 12 weeks, but the reported sleepiness was reduced, suggesting a modification in sleep patterns, even if unrecognized by the patients.

## Concluding Remarks and Future Perspectives

Circadian rhythms lie at the base of complex life, and are required for the regulation of energy metabolic processes. When peripheral clocks are led out of phase respect to the central clock, or among each other, metabolic alterations may occur. The timing of food intake, diet composition and sleep all play roles in regulating circadian rhythm.

Some research studies in animal models suggest that a ketogenic diet is able to influence circadian biology, through the modulation of clock gene expression. KD seems to have a profound impact on circadian rhythm, metabolism and behavioral activity, and induces a higher amplitude of gene expression and *de novo* oscillating genes at hepatic level. How these effects manifest, and what the long-term consequences are, remain to be determined. The role of ketosis itself is yet to be completely elucidated. At the same time, the effects of diet composition that have been studied and observed are mainly related to peripheral circadian clock regulation rather than central core clock regulation, so it is still not elucidated if nutrients may exert a direct effect on the central core clock rhythm.

Other studies with animal models and studies on circadian rhythm in humans are necessary to answer these questions which are recently born.

The interaction among diet, in particular the ketogenic diet, and circadian rhythm is extremely complex, and research at its dawn has already given intriguing results, taking potential important new horizons on obesity treatment.

## Author Contributions

EG and LG: conceptualization. EG: writing—original draft preparation. LG, AL, and DR: writing—review and editing. All authors have read and agreed to the published version of the manuscript.

## Conflict of Interest

The authors declare that the research was conducted in the absence of any commercial or financial relationships that could be construed as a potential conflict of interest.

## Publisher’s Note

All claims expressed in this article are solely those of the authors and do not necessarily represent those of their affiliated organizations, or those of the publisher, the editors and the reviewers. Any product that may be evaluated in this article, or claim that may be made by its manufacturer, is not guaranteed or endorsed by the publisher.
